# Detection of Severe Acute Respiratory Syndrome Coronavirus 2 (SARS-CoV-2) by Mass Spectrometry

**DOI:** 10.3390/v12080849

**Published:** 2020-08-04

**Authors:** Petra Wandernoth, Katharina Kriegsmann, Cristina Groh-Mohanu, Martin Daeumer, Peter Gohl, Oliver Harzer, Mark Kriegsmann, Joerg Kriegsmann

**Affiliations:** 1 Center for Histology, Cytology and Molecular Diagnostics Trier, 54296 Trier, Germany; p.wandernoth@molekularpatho-trier.de (P.W.); c.mohanu@molekularpatho-trier.de (C.G.-M.); kriegsmann@patho-trier.de (J.K.); 2 Department of Hematology, Oncology and Rheumatology, University Hospital Heidelberg, 69120 Heidelberg, Germany; katharina.kriegsmann@med.uni-heidelberg.de; 3 Kaiserslautern Medical Laboratory, Institute of Immunology and Genetics, 67655 Kaiserslautern, Germany; m.daeumer@immungenetik-kl.de; 4 Bioscientia, 576080 Ingelheim, Germany; peter.gohl@bioscientia.de (P.G.); oliver.harzer@bioscientia.de (O.H.); 5 Institute of Pathology, University Hospital Heidelberg, 69120 Heidelberg, Germany; 6 German Center for Lung Cancer Research (DZL), 69120 Heidelberg, Germany; 7 Danube Private University Krems, 3500 Krems, Austria

**Keywords:** SARS-CoV2, COVID19, mass spectrometry, severe acute respiratory syndrome coronavirus 2

## Abstract

Background: Amplification of viral ribonucleic acid (RNA) by real-time reverse transcriptase polymerase chain reaction (rRT-PCR) is the gold standard to detect severe acute respiratory syndrome coronavirus 2 (SARS-CoV-2). Since the initial outbreak, strategies to detect and isolate patients have been important to avoid uncontrolled viral spread. Although testing capacities have been upscaled, there is still a need for reliable high throughput test systems, specifically those that require alternative consumables. Therefore, we tested and compared two different methods for the detection of viral PCR products: rRT-PCR and mass spectrometry (MS). Methods: Viral RNA was isolated and amplified from oro- or nasopharyngeal swabs. A total of 22 samples that tested positive and 22 samples that tested negative for SARS-CoV-2 by rRT-PCR were analyzed by MS. Results of the rRT-PCR and the MS protocol were compared. Results: Results of rRT-PCR and the MS test system were in concordance in all samples. Time-to-results was faster for rRT-PCR. Hands-on-time was comparable in both assays. Conclusions: MS is a fast, reliable and cost-effective alternative for the detection of SARS-CoV-2 from oral and nasopharyngeal swabs.

## 1. Introduction

Severe acute respiratory syndrome coronavirus 2 (SARS-CoV-2) is a positive-sense single-stranded ribonucleic acid (RNA) virus and has been identified as the causative agent of coronavirus disease 2019 (COVID-19) disease [1]. To avoid uncontrolled viral spread, high-throughput testing and subsequent isolation of infected individuals was advised [2]. However, due to limited laboratory testing capacities, only symptomatic individuals could be tested in the beginning of the 2020 pandemic [3]. On the other hand, it has been shown that a large proportion of individuals remain asymptomatic. Therefore, a clinical definition of COVID-19 is not reliable and laboratory confirmation of SARS-CoV-2 is currently advised for confirmation [4]. As asymptomatic patients tested positive for SARS-CoV-2, the lack of test capacity resulted in a lack of knowledge of the underlying true infection risk [5,6]. Since then, test capacities have been upscaled, but there is still an urgent need for reliable and cost-effective high-throughput testing methods, specifically as regulations are currently easing and loci of rapid spread need to be detected as early as possible, to avoid uncontrolled viral spread.

Mass spectrometric (MS) techniques have largely complemented or replaced traditional methods in laboratory medicine, toxicology, microbiology as well as molecular pathology and are suitable for reliable, cost-effective and rapid detection of amplified polymerase chain reaction (PCR) products [7,8,9,10,11]. Thus, this method has a great potential to complement the current diagnostic arsenal, especially in times where a shortage of reagents may limit the application of real-time reverse transcriptase (rRT)-PCR, which is the current gold standard for the detection of SARS-CoV-2 [12].

Therefore, we established and tested a MS-based test protocol for its ability to detect SARS-CoV-2 from oral or nasopharyngeal swabs and compared the results to rRT-PCR.

## 2. Materials and Methods

### 2.1. Patient Samples and Workflow

Oral or nasopharyngeal swabs were collected from patients and transferred to the laboratory in viral transport medium. RNA isolation and rRT-PCR for SARS-CoV-2 detection were conducted at the same day (Institute for Immunology and Genetics, Kaiserslautern). A total of 22 samples that tested positive and 22 samples that tested negative were selected and tested with a SARS-CoV-2 MS assay in two independent laboratories (Institute for Immunology and Genetics, Kaiserslautern and Institute for Molecular Pathology, Trier) within two weeks after rRT-PCR. Samples were obtained from corona screening centers from two local hospitals in the period from March 14, 2020, to April 24, 2020. MS and rRT-PCR results were compared. Informed written consent has been obtained from all the patients.

### 2.2. Ribonucleic Acid Extraction

RNA from clinical samples was isolated with the chemagic viral DNA/RNA 300 Kit special H96 (cat CMG-1033-S, PerkinElmer, Waltham, MA, USA) in 96-sample batches with 300 µL sample input on the chemagic Magnetic Separation Module I instrument (PerkinElmer) in approximately 60 min. Eluted RNA was used for subsequent analysis or stored at −80.

### 2.3. Real-Time Reverse Transcriptase Polymerase Chain Reaction

Amplification of viral RNA by rRT-PCR was performed with the commercially available CE-certified virellaSARS-CoV-2 seqc rRT-PCR kit including primers and dual-labeled probes (ref G01128-32, Gerbion, Kornwestheim, Germany) using 6 µl RNA input in a total RT-PCR mix of 20 µL on a ABI 7500 instrument (Applied Biosystem, Waltham, MA, USA) according to the manufacturer’s protocols. According to the manufacturer the primers used are similar to the primers published [12]. The limit of detection (LOD) of the respective kit was 10 genome copies per reaction. The assay was designed to detect the following SARS-CoV-2 targets: the RdRP (ORF1ab) and E gene.

### 2.4. SARS-CoV-2 Mass Spectrometrical Assay

A commercially available CE-certified SARS-CoV-2 multiplex assay was performed in a 96 well plate including a one-step RT-PCR, a Shrimp-Alkaline-Phosphatase (SAP) and an extension reaction using the SARS-CoV-2 Reagent Set (ref. 13274F, Agena Bioscience, Hamburg, Germany) according to the manufacturer’s protocol R1.X1 (Agena Bioscience). Three µL RNA input in a total RT-PCR mix of 5 µL was used on a Thermocycler (Biometra TAdvanced PCR Thermocycler, Analytic Jena, Jena, Germany). To remove surplus nucleotides each RT-PCR reaction was treated with SAP mix. Two µL extension reaction mix composed of mass-modified terminator nucleotides was added to elongate the amplified complementary deoxyribonucleic acid (cDNA) strands at the nucleotide position of interest, depending on the presence of SARS-CoV-2 virus. The assay included a RNaseP target as internal quality control (QC). Each sample was treated with 41 µL nanopure water (>18 mΩ) and transferred into a Chip Prep Module (Agena Bioscience) to process post extend PCR samples for desalting in 15 mg clean resin (ref. 8040, Agena Bioscience), spotting on a matrix-precoated Spectro-CHIP (Agena Bioscience) and analysis in a matrix assisted laser desorption/ionization time-of-flight mass spectrometer (MassARRAY Analyzer 4, Agena Bioscience). A final report (SC2 Report v1.0, Agena Bioscience) was generated within the commercial software (MassARRAY Typer Analyzer Software v4.1.83, Agena Bioscience), which listed all samples, the quality control and SARS-CoV-2 status, followed by details on each individual SARS-CoV-2 target. The LOD of the respective kit was 10 genome copies per reaction. The assay was designed to detect the following SARS-CoV-2 targets: viral nucleocapsid genes (N1, 2 and 3; genome areas: 28653-28760, 28880-28978, 28076-28190), ORF1ab/nsp3 and ORF1ab/nsp10; genome areas: 3223-3335 and 13342-13432 (Table 1). All assay components exhibit 100% sequence homology to conserved SARS-CoV-2 regions except for the forward PCR primer for the SC2_N2 assay. According to the sequence data as of May 20, 2020, 8% of the SARS-CoV-2 sequences have a three-nucleotide mismatch with the first three 5′ end nucleotides of the SC2_N2 forward PCR primer (22 nucleotide length). This results in the 86% PCR primer homology for the 8% of SARS-CoV-2 population and the 98.9% weighted homology. The mismatch is located at the 5′ end of the PCR primer and does not affect the test performance [13]. Samples were identified as positive if ≥2 SARS-CoV-2 targets were detected and the QC passed, invalid if the QC failed and negative if <2 SARS-CoV-2 targets were detected and the QC passed.

### 2.5. Descriptive Statistics

Descriptive statistics were performed in R statistical software (v. 4.0.0. R Development Core Team, 2008). Data are presented as absolute numbers and percentage as well as median and range.

## 3. Results

For 43 of 44 patient samples basic clinical data were available. 30 (70%) patients were male and 13 (30%) female. Mean age was 43 (min–max: 7–77).

**Figure 1:**
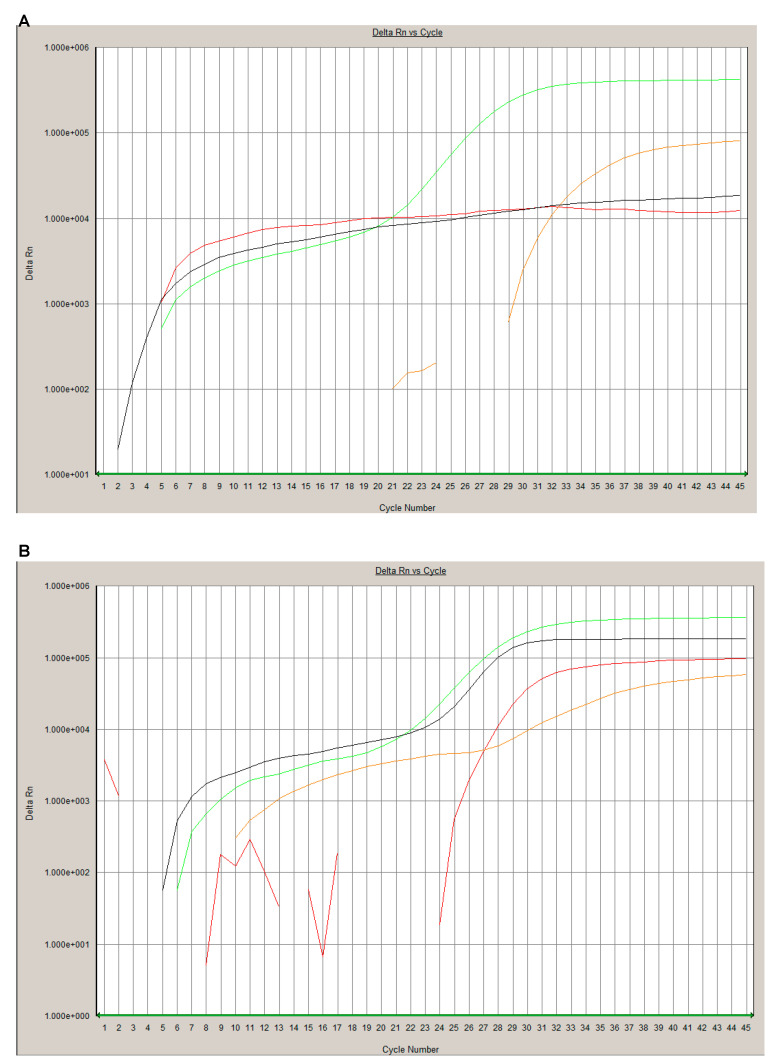
Examples for a positive and negative rRT-PCR result. A SARS-CoV-2 negative sample ((**A**); ID 17) show a signal in the control RNA specific HEX channel (orange line) and process control channel ROX channel (green line). A SARS-CoV-2 positive sample ((**B)**; ID 5) shows additionally a SARS-CoV-2 specific amplification in the FAM channel (red line) and beta-Coronavirus detection (E gene) in the Cy5 channel (black line).

**Figure 2:**
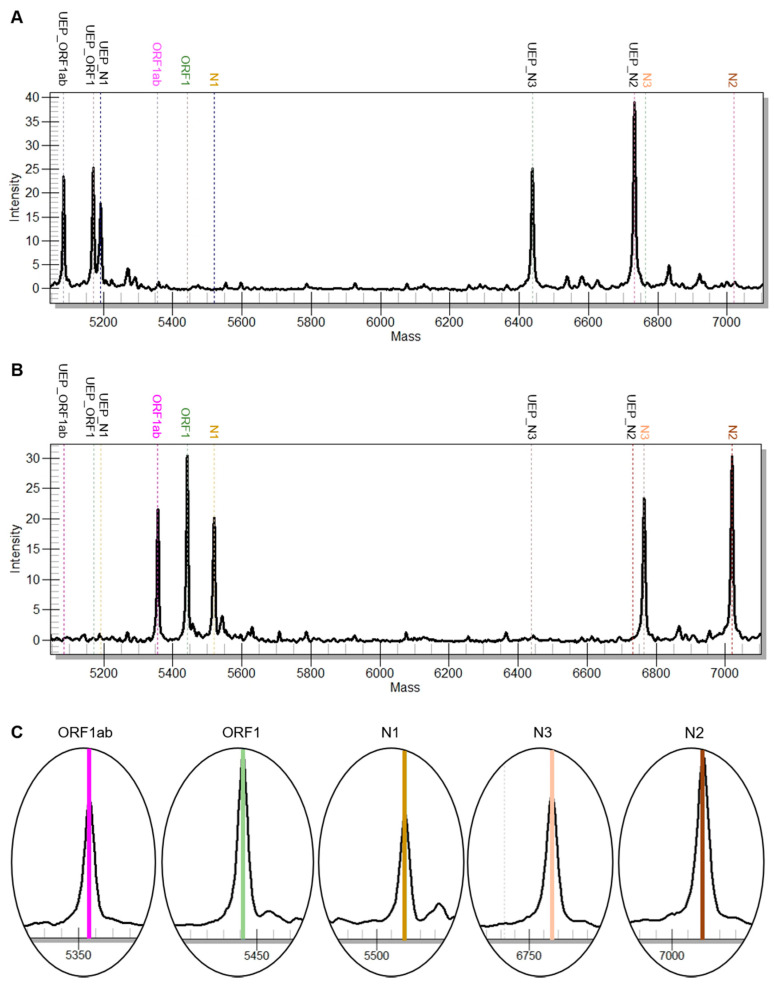
Examples for a positive and negative result of the commercial MS assay. In the sum spectra (**A**,**B**) the expected mass peaks for the respective amplicons are highlighted with a dashed line. In the negative case (**A**, ID 17) peaks of unextended primers are observed (UEP). In the positive case (**B**, ID 5) peaks for amplicons of the ORF1ab, ORF1, N1, N2 and N3 can be identified. Zoomed spectra of these amplicons are depicted in (**C**).

All samples included in the study (*n* = 44) were successfully analyzed by rRT-PCR and the MS test system. None of the 44 samples failed quality control. Positive and negative test results were concordant in all samples. A representative example of a positive and a negative result of both tests is shown in Figure 1 and Figure 2.

Time-to-results of the rRT-PCR, the MS test were 120 min and 480 min, respectively. Hands-on-time of the rRT-PCR and the MS test were 40 min for both (Table 2).

## 4. Discussion

A total of six coronaviruses are known to cause human disease from which two strains (SARS-CoV-2 and Middle East respiratory syndrome coronavirus) are thought to be zoonotic and have been associated with more severe, potentially fatal outcomes [14].

SARS-CoV-2 is an enveloped single-stranded positive-sense RNA virus and belongs to the genus of beta-Coronaviruses, is spherical in shape, about 60–140 nm in diameter and has 9–12 nm long characteristic spikes on the surface [15]. This virus has been identified as the causative agent of COVID-19 disease after the World Health Organization Country Office was informed about cases of pneumonia of unknown etiology in Wuhan City, Hubei Province in China on 31 December 2019 [12]. To detect infection with SARS-CoV-2 saliva, sputum, oral or nasopharyngeal swabs, stool, anal swabs, blood or urine can be analyzed and highest rates of detection have been reported for sputum, oral or nasopharyngeal swabs [16]. However, the peak diagnostic yield might depend on the time of onset of symptoms [17]. In this study, we tested material derived from oral and nasopharyngeal swabs.

The genome of a typical CoV contains a 5′ untranslated region (UTR); a conserved replicase domain (ORF1ab including the RdRp gene); four genes S, E, M and N to encode structural proteins spike, envelope, membrane, and nucleocapsid proteins; a 3′ UTR and several unidentified non-structural ORFs, which are potential targets for their detection [18]. These gene regions may be used to identify infection with SARS-CoV-2.

Interestingly, recommendations regarding which gene regions should be targeted to identify the virus are highly variable. For example, in the US, three genes targeting the N-gene are recommended for emergency testing; in Germany, the Charité recommends the identification of the E gene for screening and identification of the RdRp gene for confirmation [12]. The latter recommendation is based on the fact that the region of the E gene is commonly shared between SARS-CoV-2 and other bat or human related SARS viruses, and the RdRP sequence is specific for SARS-CoV-2 [12]. Generally, two to three targets need to be detected to qualify as a reliable result [18]. Moreover, an internal quality control is currently recommended, which is also in the MS detection kit [18].

The analysis of rRT-PCR may be affected by false-negative and false-positive results. In this regard, it has been shown that multiple testing in the course of disease can improve the rate of viral detection [4,16,19,20,21]. One study found that in 21.4% of patients, a positive detection of SARS-CoV-2 could only be achieved after two consecutive negative results [21]. Interestingly, 16.7% of patients with typical features of COVID-19 pneumonia on a computer tomography scan were negative by rRT-PCR; half of these patients became positive after the second test, and half of the remaining patients became positive after the third test [4]. It was found that the rRT-PCR results from several tests at different time points were variable from the same patients during the course of diagnosis and treatment [19]. This might explain the fact that only about 50% of clinically confirmed cases are confirmed by a positive rRT-PCR result. As a consequence, a negative rRT-PCR should not lead to the false assumption that the patient is not infected by SARS-CoV-2, and clinical parameters need to be considered [20,22]. To avoid false-negative results, due to testing failures, standardized collection, transport conditions, storage, extraction and amplification procedures of patient material are needed. Interestingly, thermal inactivation reduced the detectable amount of SARS-CoV-2 in rRT-PCR runs and would be expected also for our MS test. On the other hand, inactivation by guanidinium-based lysis exhibited less effects [23].

Besides rRT-PCR (gold standard), alternative techniques such as isothermal amplification methods and MS are available for the detection of viruses [24,25]. MS has been successfully used for the detection of viral DNA, RNA or proteins in previous studies [26,27,28]. The method is fast with a turnaround time of max. 1 day and therefore suitable for routine use [29]. However, to the best of our knowledge there have been no reports on the application of MS for the detection of SARS-CoV-2 to date. We show that (i) rRT-PCR is the fastest method to detect SARS-CoV-2, (ii) hands-on-time is comparable between rRT-PCR and the MS method and (iii) results are concordant between both assays.

## 5. Conclusions

In summary, we report on the application of MS as a reliable and fast method for the detection of SARS-CoV-2. Since there is a shortage of reagents at several places, alternative methods that complement rRT-PCR and are dependent on alternative reagents are highly beneficial to tackle the 2020 COVID-19 pandemic.

## Figures and Tables

**Table 1:** Targets of the MS assays.

MS Assay		
Target	Genomic Region	Mass EA (Da)
SC2_N1	N1	5519.50
SC2_N2	N2	7019.60
SC2_N3	N3	6765.40
SC2_ORF1	ORF1ab/nsp3	5441.60
SC2_ORF1ab	ORF1ab/nsp10	5356.50

Da: Dalton; EA: extended amplicon; MS: mass spectrometry; N: nucleocapsid protein gene; ORF: open reading frame.

**Table 2:** Time-to-results and hands-on-time of the rRT-PCR and the commercial MS assay.

Steps	rRT-PCR		MS Assay	
	Run Time (min)	Hands-on-Time (min)	Run Time (min)	Hands-on-Time (min)
rRT-PCR	75	30	150	15
SAP	/	/	50	5
Extensions-PCR	/	/	120	10
CPM process	/	/	90	5
Data acquisition and analysis	5	10	30	5
Overall	120		480	

CPM: Chip Prep Module; MS: mass spectrometry; rRT-PCR: real-time reverse-transcriptase polymerase chain reaction; SAP: Shrimp-Alkaline-Phosphatase.
